# Evaluation of regional antibiograms to monitor antimicrobial resistance in hampton roads, Virginia

**DOI:** 10.1186/s12941-015-0080-6

**Published:** 2015-04-09

**Authors:** Susette K Var, Rouba Hadi, Nancy M Khardori

**Affiliations:** Department of Internal Medicine, Eastern Virginia Medical School, 825 Fairfax Ave, Hofheimer Hall, Ste 572, Norfolk, VA 23507, USA

**Keywords:** Antibacterial susceptibility, Antibiogram, Antibiotic resistance, Antibiotic utilization, Antimicrobial stewardship, Antimicrobial susceptibility, Surveillance

## Abstract

We studied recent antibiograms (2010 to 2011) from 12 hospitals in the Hampton Roads area, Virginia, that refer patients to a tertiary-care facility affiliated with Eastern Virginia Medical School. The data was compiled into a regional antibiogram, and sensitivity rates of common isolates from the tertiary-care facility (central) were compared to those of referring hospitals grouped by locale. Staphylococcus aureus was the most common Gram- positive and E. coli the most common Gram- negative organism grown from clinical samples in the area. Overall 53% of S.aureus isolates were resistant to oxacillin. There was a broad scatter of MIC (minimum inhibitory concentration) for vancomycin within the susceptibility range, and MIC of 4 μg/mL was reported in 2012. Penicillin resistance was seen in 50% and erythromycin resistance in 45% of Streptococcus pneumoniae. Vancomycin resistance was seen in 75% of Enterococcus faecium and 2% of Enterococcus faecalis respectively. Acinetobacter baumannii was the most resistant Gram negative organism in the data compiled. Among the Escherichia coli, 26%, 44% and 52%were resistant to Trimethoprim/Sulfamethoxazole ( SXT) ampicillin- sulbactam and ampicillin respectively. We found significant differences in methodology, interpretation and antibiotic panels used by area laboratories. Based on these findings, we are now prospectively following resistance patterns in the tertiary-care facility, sharing data, and creating a consistent approach to antimicrobial susceptibility testing in the region.

## Background

National databases are unlikely to prove useful in making antimicrobial use in communities more appropriate. Such databases of academic and educational value are useful for microbiologists and infectious disease clinicians. In the community, however, databases are less useful because: 1) most antimicrobial drug prescriptions in the community are written by primary care physicians who generally do not use these resources, 2) industry-generated data are often used to highlight a particular antimicrobial agent, 3) there are regional differences in antimicrobial resistance patterns, which, at times, may not reflect clearly in national databases, and 4) a significant number of patients are transferred from local hospitals in the region to tertiary-care/referral hospitals. The appropriate management of serious, non-resolving infections in the transferred patients should be guided by resistance patterns prevalent in the referring hospital. Because of prior antimicrobial therapy, the repeat cultures done at the receiving hospital often do not yield helpful results.

To date, regional studies concerning antimicrobial resistance patterns in the Hampton Roads area of Virginia, a region with a diverse and a high population density have been limited [1]. More common are studies that concentrate on one particular organism or group of organisms and its resistance patterns in the United States by regions encompassing larger areas, such as “East North Central” and Mid-Atlantic regions or entire states in the United States [2-4]. These studies had the benefit of the Surveillance and Control of Pathogens of Epidemiological Importance (SCOPE)—MediMedia Information Technology (MMIT) Antimicrobial Monitoring Network. This network surveyed nosocomial infections across the United States and is based at Virginia Commonwealth University in Richmond, Virginia [5]. The database provided access to a vast amount of data that also was linked to specific subjects, but it since ended its activities in 2004 [5]. Aside from this database, the Centers for Disease Control and Prevention (CDC) publish its own reports that trace antibiotic susceptibilities across the United States [6]. However, this is national data, which is useful for comparisons and for surveillance but not so much for treatment guidance at the regional and local levels. The aim of this retrospective study was to compile the region-wide antibiotic resistance data and categorize it by central (tertiary care) and the referring hospitals by locale. The availability of a regional antibiogram allows for dissemination and sharing of prevailing resistance patterns And selection of more appropriate antimicrobial therapy at the referring as well as tertiary care hospitals.

## Methods

Requests for the two most-recent antibiograms were sent to microbiology laboratories of the sixteen hospitals that refer patients to a tertiary-care center in the region of southeastern Virginia known as Hampton Roads. The latest available antibiograms were for the years 2010 and 2011. Seventy-five percent of the referring hospitals responded. These hospitals reflected the variety of locales served in the area, for not only does the tertiary-care facility receive patients from Hampton Roads, but also transfers from hospitals in western Virginia and northern North Carolina. In essence, patients from seventeen hospitals within a seventy-mile radius converge are sent to this one “central” hospital.

Data from a total of twelve responding hospitals along with data from the central hospital (n = 13) were combined into a regional antibiogram. The region was divided arbitrarily into four geographical areas with the tertiary-care facility at the central point. The northwest region included 3 hospitals, northeast 4, southwest 4, and southeast 1 hospital. The antibiotics presented in this paper are ones tested at the central hospital since the testing panels used were different for various hospitals. The referring hospitals may test more or less antibiotics on particular isolates. This manuscript is based on a summary of diverse experimental procedures used by the microbiology laboratories from the participating hospitals. The P values were derived by unpaired T-tests using an online calculator.

## Results and discussion

Overall, *Staphylococcus aureus* (19,515 isolates) was the most common organism isolated, followed closely by *Escherichia coli* (18,812). Coagulase-negative staphylococci, *Klebsiella pneumoniae, Pseudomonas aeruginosa,* and *Enterobacter* species were among the next five most frequently reported organisms (Tables 1 and 2).

**Table 1:** **Susceptibility results of common Gram-positive bacteria to select antimicrobial agents**

**% isolates susceptible (no. isolates reported)**																
Organism/region (no. isolates tested)	AMP	CFZ	CTX	CLI	DAP	ERY	GEN	LZD	MXF	NIT	OXA	PEN	Q-D	TET	SXT	VAN
*Staphylococcus aureus*																
All (19,515)	-	46 (18,727)	-	72 (19,253)	100 (7,041)	-	98 (12,956)	100 (11,037)	-	-	47 (18,727)	-	-	96 (19,315)	99 (19,312)	100 (18,890)
Central (3,602)	-	49 (3,602)	-	72 (3,402)	100 (1,712)	-	99 (1,712)	100 (1,712)	-	-	49 (3,602)	-	-	96 (3,402)	99 (3,402)	100 (3,402)
Northwest (3,883)	-	45 (3,883)	-	70 (3,883)	100 (1,531)	-	98 (3,883)	100 (2,710)	-	-	44 (3,883)	-	-	94 (3,883)	98 (3,883)	100 (3,883)
Northeast (5,389)	-	48 (5,045)	-	67 (5,327)	100 (2,995)	-	97 (5,045)	100 (4,725)	-	-	48 (5,045)	-	-	97 (5,389)	99 (5,386)	100 (5,389)
Southwest (6,246)	-	45 (5,802)	-	79 (6,246)	100 (576)	-	98 (1,921)	100 (1,495)	-	-	46 (5,802)	-	-	96 (6,246)	98 (6,246)	100 (5,821)
Southeast (395)	-	42 (395)	-	69 (395)	100 (227)	-	98 (395)	100 (395)	-	-	42 (395)	-	-	94 (395)	97 (395)	100 (395)
*Coagulase-negative staphylococci*																
All (13,289)	-	38 (12,784)	-	64 (13,160)	-	-	85 (9,644)	-	-	-	39 (12,143)	-	-	83 (11,833)	-	100 (13,237)
Central (3,043)	-	33 (3,043)	-	58 (3,043)	-	-	82 (1,332)	-	-	-	33 (3,043)	-	-	82 (3,043)	-	100 (3,043)
Northwest (2,420)	-	41 (2,420)	-	62 (2,420)	-	-	85 (2,420)	-	-	-	42 (2,038)	-	-	83 (2,420)	-	100 (2,420)
Northeast (4,764)	-	42 (4,475)	-	66 (4,635)	-	-	86 (4,476)	-	-	-	42 (4,379)	-	-	83 (4,764)	-	100 (4,764)
Southwest (2,548)	-	35 (2,332)	-	69 (2,548)	-	-	83 (902)	-	-	-	38 (2,169)	-	-	84 (1,092)	-	100 (2,496)
Southeast (514)	-	45 (514)	-	72 (514)	-	-	89 (514)	-	-	-	45 (514)	-	-	81 (514)	-	100 (514)
*Streptococcus pneumoniae*																
All (2,294)	-	-	91 (706)	-	-	55 (972)	-	-	99 (803)	-	-	50 (2,294)	-	75 (813)	-	100 (956)
Central (195)	-	-	87 (186)	-	-	54 (186)	-	-	97 (102)	-	-	52 (195)	-	79 (186)	-	100 (186)
Northwest (430)	-	-	92 (169)	-	-	57 (190)	-	-	100 (169)	-	-	31 (430)	-	74 (190)	-	100 (216)
Northeast (429)	-	-	96 (278)	-	-	64 (314)	-	-	100 (278)	-	-	50 (429)	-	75 (314)	-	100 (230)
Southwest (1,044)	-	-	88 (41)	-	-	41 (250)	-	-	97 (222)	-	-	66 (1,044)	-	71 (91)	-	100 (292)
Southeast (196)	-	-	81 (32)	-	-	65 (32)	-	-	100 (32)	-	-	12 (196)	-	82 (32)	-	100 (32)
Organism/region (no. isolates tested)	AMP	CFZ	CTX	CLI	DAP	ERY	GEN	LZD	MXF	NIT	OXA	PEN	Q-D	TET	SXT	VAN
*Enterococcus faecalis*																
All (4,390)	97 (4,254)	-	-	-	-	-	-	99 (2,914)	-	97 (3,966)	-	97 (4,174)	-	-	-	98 (4,390)
Central (777)	98 (777)	-	-	-	-	-	-	100 (413)	-	99 (413)	-	98 (777)	-	-	-	98 (777)
Northwest (1,200)	97 (1,200)	-	-	-	-	-	-	100 (604)	-	99 (1,200)	-	97 (1,200)	-	-	-	98 (1,200)
Northeast (1,391)	98 (1,255)	-	-	-	-	-	-	99 (1,100)	-	99 (1,331)	-	98 (1,255)	-	-	-	98 (1,391)
Southwest (874)	97 (874)	-	-	-	-	-	-	98 (649)	-	91 (874)	-	97 (794)	-	-	-	99 (874)
Southeast (148)	98 (148)	-	-	-	-	-	-	98 (148)	-	99 (148)	-	98 (148)	-	-	-	100 (148)
*Enterococcus faecium*																
All (1,090)	10 (1,072)	-	-	-	-	-	-	98 (951)	-	-	-	10 (1,057)	99 (951)	-	-	25 (1,089)
Central (248)	8 (248)	-	-	-	-	-	-	100 (109)	-	-	-	8 (248)	100 (109)	-	-	22 (248)
Northwest (257)	9 (257)	-	-	-	-	-	-	97 (257)	-	-	-	9 (257)	99 (257)	-	-	30 (257)
Northeast (441)	11 (363)	-	-	-	-	-	-	98 (441)	-	-	-	10 (424)	98 (441)	-	-	23 (441)
Southwest (105)	13 (31)	-	-	-	-	-	-	96 (105)	-	-	-	11 (89)	99 (105)	-	-	25 (104)
Southeast (39)	18 (39)	-	-	-	-	-	-	97 (39)	-	-	-	18 (39)	100 (39)	-	-	33 (39)

*Abbreviations*: AMP ampicillin, CFZ cefazolin, CTX cefotaxime, CLI clindamycin, DAP daptomycin, ERY erythromycin, GEN gentamicin, LZD linezolid, MXF moxifloxacin, NIT nitrofurantoin, OXA oxacillin, PEN penicillin, Q-D quinupristin/dalfopristin, TET tetracycline, SXT trimethoprim/sulfamethoxazole, VAN vancomycin.

**Table 2:** **Susceptibility results of common Gram-negative bacteria to select antimicrobial agents**

**% isolates susceptible (no. isolates reported)**																
Organism/region (no. isolates tested)	AMK	AMP	SAM	AZM	CFZ	FEP	CFT	CIP	GEN	MEM	MXF	NIT	TZP	TGC	TOB	SXT
*Escherichia coli*																
All (18,812)	99 (18,475)	48 (18,475)	56 (18,475)	93 (11,881)	87 (14,583)	95 (18,475)	94 (18,524)	70 (17,614)	88 (18,475)	100 (13,780)	71 (8,138)	90 (17,749)	94 (17,277)	99 (14,500)	90 (17,277)	74 (18,812)
Central (2,195)	100 (2,195)	47 (2,195)	58 (2,195)	93 (2,195)	85 (2,195)	94 (2,195)	92 (2,195)	65 (2,195)	89 (2,195)	100 (2,195)	69 (2,195)	93 (2,195)	93 (2,195)	100 (2,195)	90 (2,195)	73 (2,195)
Northwest (3,291)	100 (3,291)	47 (3,291)	58 (3,291)	95 (3,291)	88 (2,851)	95 (3,291)	94 (3,291)	69 (2,690)	87 (3,291)	100 (2,851)	72 (2,082)	88 (3,291)	91 (2,690)	98 (2,082)	90 (2,690)	77 (3,291)
Northeast (4,919)	99 (4,919)	49 (4,919)	58 (4,919)	93 (4,919)	85 (4,919)	94 (4,919)	92 (4,919)	64 (4,653)	90 (4,919)	100 (4,296)	63 (3,096)	85 (4,770)	93 (4,653)	100 (2,970)	88 (4,653)	72 (4,919)
Southwest (8,824)	99 (8,487)	49 (8,487)	54 (8,487)	93 (1,893)	88 (4,595)	97 (8,487)	96 (8,536)	76 (8,493)	87 (8,487)	99 (4,785)	95 (1,182)	93 (7,910)	96 (8,156)	99 (7,670)	90 (8,156)	75 (8,824)
Southeast (496)	99 (496)	51 (496)	62 (496)	93 (496)	96 (496)	89 (496)	95 (496)	89 (496)	74 (496)	100 (126)	95 (496)	79 (126)	90 (126)	95 (496)	98 (496)	93 (496)
*Pseudomonas aeruginosa*																
All (5,317)	94 (5,317)	-	-	63 (3,719)	-	80 (5,129)	-	71 (4,666)	78 (5,317)	86 (4,709)	-	-	86 (4,704)	-	94 (4,704)	-
Central (951)	96 (951)	-	-	63 (951)	-	80 (951)	-	76 (951)	81 (951)	86 (951)	-	-	86 (951)	-	96 (951)	-
Northwest (757)	97 (757)	-	-	67 (757)	-	84 (649)	-	65 (507)	78 (757)	89 (649)	-	-	83 (507)	-	90 (507)	-
Northeast (1,483)	94 (1,483)	-	-	62 (1,483)	-	78 (1,483)	-	64 (1,387)	76 (1,483)	84 (1,427)	-	-	83 (1,387)	-	92 (1,387)	-
Southwest (2,106)	93 (2,106)	-	-	63 (508)	-	80 (2,004)	-	77 (1,779)	77 (2,106)	88 (1,640)	-	-	91 (1,817)	-	95 (1,817)	-
Southeast (142)	96 (142)	-	-	64 (142)	-	84 (56)	-	75 (56)	77 (142)	89 (56)	-	-	83 (56)	-	95 (56)	-
*Proteus mirabilis*																
All (4,566)	100 (4,511)	83 (4,511)	90 (4,511)	89 (3,357)	86 (3,734)	90 (4,511)	91 (4,537)	69 (4,189)	93 (4,511)	100 (3,980)	64 (2,315)	-	97 (3,878)	-	96 (3,878)	76 (4,566)
Central (641)	100 (641)	88 (641)	89 (641)	88 (641)	84 (641)	85 (641)	85 (641)	65 (641)	98 (641)	100 (641)	65 (641)	-	97 (641)	-	98 (641)	74 (641)
Northwest (725)	100 (725)	84 (725)	90 (725)	93 (725)	91 (644)	91 (725)	91 (725)	70 (580)	91 (725)	100 (725)	73 (448)	-	99 (499)	-	95 (499)	75 (725)
Northeast (1,442)	100 (1,442)	72 (1,442)	89 (1,442)	85 (1,442)	80 (1,442)	83 (1,442)	85 (1,442)	56 (1,358)	95 (1,442)	100 (1,236)	50 (999)	-	95 (1,358)	-	97 (1,358)	67 (1,442)
Southwest (1,840)	99 (1,785)	88 (1,785)	91 (1,785)	94 (631)	93 (1,086)	98 (1,785)	97 (1,811)	80 (1,692)	91 (1,785)	100 (1,538)	97 (309)	-	99 (1,459)	-	95 (1,459)	84 (1,840)
Southeast (107)	100 (107)	72 (107)	86 (107)	76 (107)	80 (29)	74 (107)	75 (107)	66 (107)	66 (107)	100 (29)	90 (107)	-	100 (29)	-	100 (29)	92 (107)
Organism/region (no. isolates tested)	AMK	AMP	SAM	AZM	CFZ	FEP	CFT	CIP	GEN	MEM	MXF	NIT	TZP	TGC	TOB	SXT
*Klebsiella pneumoniae*																
All (6,848)	93 (6,806)	-	79 (6,806)	84 (5,087)	84 (5,895)	88 (6,806)	88 (6,840)	87 (6,435)	94 (6,806)	93 (5,627)	79 (3,957)	-	89 (6,259)	94 (5,530)	89 (6,393)	85 (6,848)
Central (1,422)	90 (1,275)	-	75 (1,275)	87 (1,275)	81 (1,275)	89 (1,275)	88 (1,275)	85 (1,275)	96 (1,275)	94 (1,275)	86 (1,275)	-	89 (1,275)	96 (1,275)	87 (1,275)	84 (1,275)
Northwest (1,101)	97 (1,101)	-	84 (1,101)	91 (1,101)	88 (906)	91 (1,101)	91 (1,101)	90 (946)	97 (1,101)	98 (906)	91 (839)	-	93 (751)	96 (839)	93 (946)	90 (1,101)
Northeast (1,687)	89 (2,204)	-	75 (2,204)	81 (2,204)	78 (2,204)	83 (2,204)	81 (2,204)	80 (2,094)	89 (2,204)	88 (2,052)	71 (1,666)	-	86 (2,094)	92 (1,618)	81 (2,094)	81 (2,204)
Southwest (2,414)	97 (2,372)	-	84 (2,372)	84 (653)	91 (1,595)	94 (2,372)	94 (2,406)	94 (2,266)	96 (2,372)	96 (1,479)	80 (323)	-	91 (2,224)	95 (1,944)	97 (2,224)	89 (2,414)
Southeast (224)	84 (224)	-	68 (224)	79 (224)	95 (90)	77 (224)	82 (224)	80 (224)	79 (224)	97 (90)	84 (224)	-	100 (90)	82 (224)	94 (224)	80 (224)
*Serratia marcescens*																
All (991)	99 (954)	-	-	97 (632)	-	99 (917)	97 (954)	93 (971)	97 (954)	99 (863)	-	-	91 (917)	96 (920)	93 (954)	98 (971)
Central (319)	99 (319)	-	-	98 (319)	-	99 (319)	97 (319)	91 (319)	99 (319)	100 (319)	-	-	93 (319)	96 (319)	98 (319)	99 (319)
Northwest (65)	100 (65)	-	-	99 (65)	-	100 (65)	99 (65)	92 (65)	100 (65)	100 (65)	-	-	100 (65)	100 (65)	99 (65)	95 (65)
Northeast (231)	100 (231)	-	-	96 (231)	-	99 (231)	96 (231)	93 (231)	97 (231)	99 (227)	-	-	94 (231)	96 (197)	93 (231)	99 (231)
Southwest (376)	100 (359)	-	-	89 (37)	-	98 (322)	97 (359)	95 (376)	96 (359)	99 (272)	-	-	86 (322)	95 (359)	87 (359)	96 (376)
Southeast (0)	-	-	-	-	-	-	-	-	-	-	-	-	-	-	-	-
*Enterobacter aerogenes*																
All (824)	100 (790)	-	-	83 (493)	-	99 (763)	83 (493)	93 (802)	99 (790)	99 (657)	-	-	81 (763)	97 (749)	99 (790)	96 (802)
Central (216)	100 (216)	-	-	84 (216)	-	99 (216)	84 (216)	95 (216)	99 (216)	99 (216)	-	-	76 (216)	96 (216)	99 (216)	97 (216)
Northwest (69)	100 (69)	-	-	82 (69)	-	100 (69)	79 (69)	94 (69)	100 (69)	100 (69)	-	-	73 (69)	96 (69)	100 (69)	97 (69)
Northeast (203)	100 (203)	-	-	82 (203)	-	100 (203)	82 (203)	88 (203)	98 (203)	99 (186)	-	-	78 (203)	99 (162)	98 (203)	93 (203)
Southwest (336)	100 (324)	-	-	85 (27)	-	99 (297)	100 (27)	96 (336)	99 (324)	100 (208)	-	-	89 (297)	97 (324)	99 (324)	97 (336)
Southeast (0)	-	-	-	-	-	-	-	-	-	-	-	-	-	-	-	-
Organism/region (no. isolates tested)	AMK	AMP	SAM	AZM	CFZ	FEP	CFT	CIP	GEN	MEM	MXF	NIT	TZP	TGC	TOB	SXT
*Enterobacter cloacae*																
All (1,795)	100 (1,773)	-	-	78 (1,293)	-	96 (1,711)	77 (1,295)	86 (1,692)	95 (1,773)	99 (1,551)	86 (988)	-	76 (1,608)	93 (1,462)	95 (1,670)	87 (1,795)
Central (388)	100 (388)	-	-	80 (388)	-	98 (388)	80 (388)	93 (388)	98 (388)	100 (388)	95 (388)	-	82 (388)	96 (388)	98 (388)	96 (388)
Northwest (350)	100 (350)	-	-	66 (350)	-	95 (324)	64 (350)	62 (309)	89 (350)	99 (324)	66 (247)	-	49 (283)	87 (247)	87 (309)	77 (350)
Northeast (456)	100 (456)	-	-	82 (456)	-	98 (456)	81 (456)	89 (427)	96 (456)	100 (436)	89 (342)	-	84 (427)	94 (316)	96 (427)	85 (456)
Southwest (627)	100 (605)	-	-	84 (125)	-	94 (568)	91 (127)	92 (594)	95 (605)	99 (428)	100 (37)	-	78 (535)	94 (537)	96 (572)	88 (627)
Southeast (65)	100 (25)	-	-	80 (25)	-	-	96 (25)	84 (25)	96 (25)	-	96 (25)	-	-	80 (25)	88 (25)	100 (25)
*Acinetobacter baumannii*																
All (1,100)	67 (505)	-	75 (870)	-	-	40 (823)	-	40 (734)	51 (1,045)	50 (737)	-	-	39 (71)	-	63 (884)	51 (1,058)
Central (222)	50 (126)	-	77 (222)	-	-	35 (222)	-	36 (222)	50 (222)	47 (222)	-	-	-	-	57 (222)	49 (222)
Northwest (144)	65 (72)	-	66 (144)	-	-	45 (144)	-	45 (108)	60 (144)	82 (108)	-	-	41 (36)	-	88 (108)	55 (144)
Northeast (409)	56 (96)	-	77 (409)	-	-	27 (246)	-	20 (201)	42 (409)	39 (333)	-	-	11 (20)	-	48 (364)	41 (409)
Southwest (279)	84 (211)	-	66 (144)	-	-	57 (211)	-	63 (203)	70 (233)	79 (37)	-	-	73 (15)	-	82 (190)	68 (246)
Southeast (46)	-	-	78 (37)	-	-	-	-	-	8 (37)	38 (37)	-	-	-	-	-	48 (37)

*Abbreviations*: *AMK* amikacin, *AMP* ampicillin, *SAM* ampicillin/sulbactam, *AZM* aztreonam, *CFZ* cefazolin, *FEP* cefepime, *CFT* ceftriaxone, *CIP* ciprofloxacin, *GEN* gentamicin, *IMI* imipenem, *MEM* meropenem, *MXF* moxifloxacin, *NIT* nitrofurantoin, *TZP* piperacillin/tazobactam, *TGC* tigecycline, *TOB* tobramycin, *SXT* trimethoprim/sulfamethoxazole.

Oxacillin resistance was demonstrated in 53% of *S. aureus* with the range in different areas being 51% to 58% (susceptibility of 42% to 49%, P < 0.0001).This was in comparison to oxacillin resistance rates of 44% to 58% reported by National Health Care safety Network (NHSN) for 2009 -2010. All tested isolates were susceptible to vancomycin, daptomycin, and linezolid. Because of differences in methodology and interpretation, we were able to determine vancomycin intermediate resistance only at limited locations. There was a broad scatter of MIC with vancomycin that was within the susceptibility range, and MIC of 4 μg/mL was reported in the year 2012. Additionally with *S. aureus*, susceptibility range was 97% to 99% for trimethoprim-sulfamethoxazole (SXT), 94% to 97% for tetracycline (P < 0.0001), and 69% to 79% for clindamycin (P < 0.0001).

Only 39% of coagulase-negative staphylococci were susceptible to oxacillin. Among *Enterococcus faecalis*, 97% were susceptible to ampicillin and 98% to vancomycin. Ampicillin and vancomycin susceptibility of *Enterococcus faecium* were 10% and 25%, respectively compared to 625 to 93% resistance to Vancomycin reported by NHSN. Susceptibility of *Streptococcus pneumoniae* in the region varied between 12% and 66% for penicillin and 41% and 65% for erythromycin.

For *E. coli*, meropenem was the most active beta-lactam (susceptibility 99% to 100%) and amikacin was the most active aminoglycoside overall (99% to 100%). Overall, 29% and 30% of E.coli were resistant to moxifloxacin and ciprofloxacin respectively. NHSN (2009-2010 ) reported 25% to 42% E.coli to be resistant to quinolones. The difference in the susceptibility of *E. coli* to ampicillin and to ampicillin/sulbactam was statistically significant at 48% and 56%, respectively (P < 0.0001).

Meropenem and amikacin were the most effective agents against *K. pneumoniae*, *Enterobacter aerogenes,* and *Enterobacter cloacae*.

*Acinetobacter baumannii* data differed widely across the regions. Susceptibility to amikacin ranged from 50% (126 isolates) to 84% (211) with 67% (505) for all reporting hospitals. The susceptibilty range for gentamicin was 8% (37) to 70% (233) with 51% (1,045) for all reporting hospitals. And for ampicillin/sulbactam, the susceptibility range was 66% (144) to 78% (37) with 75% (870) overall.

The increased prevalence of antibiotic resistant strains is associated with greater morbidity, mortality, and healthcare cost [7]. Thus, it is advantageous to have a sense of resistance patterns in a region in order to decide on the best antibiotic for use, lessening the drain on time and resources from increased lengths of stay, multiple trials of antibiotics, and/or non-judicious use of broad-spectrum antibiotics [8]. Unfortunately, the trend is towards increased use of broad-spectrum antimicrobials, because of their relative ease of use [9]. However, the threat of developing resistance to what is seen as the current last line of defense against disease-causing microbes is very real, as seen with the rare but documented vancomycin-resistant *Staphylococcus aureus* (VRSA) and the now notorious carbapenem-resistant *Enterobacteriaceae* (CRE) [10,11]. Therefore the prudent and judicious use of available agents at this time is absolutely necessary [12]. Resistance can develop and spread quickly as seen with carbapenem resistance mediated by New Delhi metallo-β-lactamase, which was documented in 2006 and then spread to five continents over a span of less than ten years [13]. There is plenty of interest in antimicrobial stewardship but the optimization of antimicrobial use has not been achieved even with the availability of national data bases [14].

One of the limitations with national publications is that they tend to focus on hospital-acquired pathogens, which typically are resistant to more antibiotics compared to community-acquired infections. Then again, data from many hospital antibiograms, including the ones used in our study, are a conglomerate of isolates from patients coming from the community and of those already in a healthcare setting, either at the central hospital or transferred from other facilities. This observation also complicates how we survey regional trends. A majority of the time, susceptibility data to measure regional trends comes from hospital-associated laboratories. It is cost-effective and easier to review data that has been compiled already, and there is little practicality in collecting specimens from the community for a battery of susceptibility testing when the resources are limited. The use of hospital antibiograms, however, likely overestimates resistance in the community. Patients who are more ill tend to be ones treated in-facility as opposed to patients with less-serious infections who receive prescriptions for antibiotics on an outpatient basis. Patients with more resistant infections tend to have samples collected for culture [15]. And an often overlooked factor is that hospitals may serve populations that are different from the community surrounding it [16]. Even with these challenges, CDC states that antibiograms can be used to monitor regional trends and to alert us to increasing resistance in an area [16].

There are a few studies that have focused on regional resistance patterns. They demonstrated geographical variations in antimicrobial susceptibilities, emphasizing the utility of local surveillance as compared with national data. Much fewer studies examined smaller, more defined areas, which have their own resistance patterns as well. For instance, the Delmarva region of the eastern United States harbors more gentamicin-resistant *Escherichia coli* due to the ubiquitous poultry industry compared to the remainder of the Chesapeake Bay region [17]. Even hospitals have their own trends with higher rates of resistance manifesting in infections acquired in intensive care units compared to nosocomial infections from other units in the same hospital [18].

Our study is modeled after a previous study published in 2005 in the central Illinois area that requested and compiled antimicrobial resistance data from hospitals that referred patients to a tertiary-care center [8]. We examined resistance patterns in the region as generated by in-house antibiograms and derived a regional antibiogram which was shared with all participating institutions. Despite the limitations of hospital antibiograms, this tailored antibiogram was more relevant to healthcare in the area compared to national data bases and antibiograms for individual hospitals and to specific antibiograms for individual hospitals. National databases are too broad for the regional variability in bacterial resistance, and hospital-specific antibiograms are not broad enough to encompass the network that is the healthcare system. We also will discuss susceptibility patterns of commonly-isolated organisms and attempted to examine evidence of multidrug resistance, which is intermediate sensitivity or resistance to at least one antimicrobial agent in at least three specific classes according to the National Healthcare Safety Network [6].

Variable patterns of sensitivities do exist within the Hampton Roads region. This is not surprising with different patient populations, conditions, environments, and antibiotic preferences within the area. However, dramatic swings in sensitivities seen especially with *A. baumannii* and *E. cloacae* may be due to the small number of isolates tested as opposed to actual large differences by locales. Because of the geography-based method we used to group the hospitals, the Southeast region was composed of one hospital. The small number of isolates from that region predictably resulted in substantial swings in susceptibility data.

As for multidrug resistance of regional isolates, interpretation is limited due to the reasons aforementioned, along with the current inability to identify intermediate resistance, to distinguish duplicate samples, and to differentiate between community-acquired and nosocomial pathogens on a majority of the antibiograms from outside institutions. However, even with these challenges, some discussion of the results is warranted. *S. aureus* at one of the sites in our study had been vancomycin sensitive until an MIC of 4 μg/mL was reported in 2012 (data not shown). According to the Clinical and Laboratory Standards Institute, this MIC puts these *S. aureus* isolates in the intermediate-sensitivity range, which spans 4 to 8 μg/mL [19]. An MIC of ≥ 4 μg/mL is concerning enough that CDC recommends retesting, and if confirmed, notification of infection control, the health department, and CDC. Unfortunately, the trend of decreased *susceptibility* of S.aureus to vancomycin is not uncommon. Increasing MICs can be seen in tertiary-care centers across the United States [20,21].

The resistance pattern of *S. pneumoniae* deserves special attention because of the magnitude of outpatient antibiotic use for presumed bacterial respiratory tract infections. Link-Gelles, et al., studied geographic and temporal trends in antimicrobial susceptibility of *S. pneumoniae* in the United States [22]. The data from eight states during 2002 to 2006 and 2007 to 2009 were reported. Unfortunately, Virginia was not one of the states included. The resistance rates to penicillin ranged from 15% to 31% in first time frame and 12% to 29% in the second. Our study in Hampton Roads, Virginia, during 2010 to 2011 shows penicillin resistance in 34% to 88% of isolates. Resistance to erythromycin during the same time period was 35% to 59%. This information is being used by the antibiotic stewardship team for two major hospital groups in the area.

With *E. coli*, there is evidence of extended-spectrum β-lactamase (ESBL) production as seen with reduced susceptibility to third- and fourth-generation cephalosporins, ceftriaxone and cefepime, respectively. ESBLs are not uncommon nowadays, and *E. coli* that produce ESBL tend to be resistant to a number of other antimicrobials such as quinolones and sulfonamides, although resistance to these antimicrobials is more common than resistance to the later generation of cephalosporins [23]. The aminoglycoside amikacin and the carbapenem meropenem showed high activity against the *E. coli* isolates, staving off the fear of significant carbapenem resistance lurking in the Hampton Roads region for now. *A. baumannii*, an infamously difficult pathogen to manage, shows reduced susceptibility to recommended agents, such as ampicillin/sulbactam, meropenem, amikacin, and tobramycin. Although some of the isolates are resistant to these antimicrobials, they may be susceptible to other agents in the same group, for example, resistance to meropenem does not mean resistance to imipenem necessarily [24]. However, many of the hospitals tested a small selection of agents and typically one agent from each class. This highlights why careful and thorough susceptibility testing is important for optimal treatment, especially with an organism like *A. baumannii*.

There are several limitations with this study. Foremost is the inherent inconsistency when requesting susceptibility data from a number of different laboratories. Unlike the studies done by Karlowsky and colleagues on the susceptibility of specific organisms [2,25], which had the advantage of having samples sent to one commercial laboratory, our study was dependent on individual hospital microbiology laboratories. Not only can testing methods differ secondary to protocol and to staff training, but antibiograms can be formulated variably, a phenomenon noted in a study by Pakyz et al. [5]. In central Illinois, our group found that a number of laboratories were not aware of the guidelines concerning antibiogram analysis and presentation published by the Clinical and Laboratory Standards Institute, the M39-A guidelines [8]. A nationwide study by Zapantis et al. found that as of 2002, 85% of the 209 institutions that participated in their study were compliant with at least half of ten M39-A guidelines [26]. The latest guideline is M39-A3, published in 2009 [27]. To date, there have been no published studies that examine adherence. The hope is that since then, compliance to the guidelines has improved. However, it is difficult for institutions with limited resources to do so, since it can take a significant amount of manpower and of time to conduct a review of isolate origin, laboratory practices, and data interpretation. Zapantis’ study also found that 14% of the antibiograms reviewed showed susceptibility results that should have alerted microbiology personnel to further review [26]. Without microbiological personnel oversight of the data generated by automated testing methods, publishing an antibiogram simply would be another task made to meet a nominal standard devoid of clinical utility.

Another possible limitation with this study is isolate anonymity since we used an already formulated data base. The specimen source and patient profile were not incorporated. Karlowsky’s studies had the advantage of data connected to a sample, which included the patient’s age, sex, and specimen source [2,25]. These nuances may narrow organism susceptibility to specific situations, but it can be argued whether this additional information may be too meticulous for the purposes of creating an aggregate antibiogram for our region. Despite these limitations, the data represent a current assessment of the prevalence of antibiotic resistance in the region in which patients are shared and transferred between health care facilities. To make the data less confusing and more user friendly , it was divided into all , central ( tertiary care referral hospital ) and four surrounding areas since we did not see any significant differences in resistance patterns between facilities within those four areas. The Tables 1 and 2 have been formulated the same way as the facility antibiograms.

With the known serious shortcomings in susceptibility testing due to variations in methodology, selection of panels, interpretation, and reporting at the local regional and national levela, we believe that cooperation and collaboration at the regional level may be the most useful public health measure towards optimizing the role of diagnostic microbiology in managing infections and antimicrobial resistance. We are in the process of formalizing a regional advisory group with microbiology laboratory personnel from all participating hospitals. This will allow for greater ease of sharing data and standardization for more appropriate comparisons. A master antibiogram for the region will allow the tertiary care institution to have information on resistance patterns in the referring hospitals which will guide antibiotic changes upon transfer. Updating the regional antibiogram once a year will allow determination of changes in resistance patterns and early recognition of “endemic resistance” based on which interventions to reduce the impact on patient care can be implemented.

## Conclusions

The institutional antibiograms generated by the hospital diagnostic laboratories are readily accessible and inexpensive tool to monitor antimicrobial resistance patterns in communities and regions. A regional antibiogram including hospitals that transfer patients between them would allow appropriate antimicrobial selection at transfer.

## Abbreviations

MIC: Minimum inhibitory concentration
SXT: Trimethoprim/Sulfamethoxazole
CDC: Center for Disease Control and Prevention
VRSA: Vancomycin Resistant S. aureus
CRE: Carbapenem Resistant Enterobacteriaceae
